# The correlation between proinsulin, true insulin, proinsulin: True insulin ratio, 25(OH) D3, waist circumference and risk of prediabetes in Hainan Han adults

**DOI:** 10.1371/journal.pone.0238095

**Published:** 2020-09-03

**Authors:** Huibiao Quan, Tuanyu Fang, Leweihua Lin, Lu Lin, Qianying Ou, Huachuan Zhang, Kaining Chen, Zhiguang Zhou

**Affiliations:** 1 Department of Metabolism & Endocrinology, The Second Xiangya Hospital, Central South University, Changsha, Hunan, China; 2 Department of Endocrinology, Hainan Affiliated Hospital of Hainan Medical University, Haikou, Hainan, China; Beijing Key Laboratory of Diabetes Prevention and Research, CHINA

## Abstract

**Purpose:**

Diabetes mellitus is a kind of highly prevalent chronic disease in the world. The intervention measures on the risk factors of prediabetes contribute to control and reduce the occurrence of diabetes. This study aimed to investigate the correlation between proinsulin (PI), true insulin (TI), PI/TI, 25(OH) D3, waist circumference (WC), and risk of prediabetes.

**Methods:**

In this cross-sectional study, 1662 subjects including 615 prediabetes and 1047 non-prediabetes were recruited. Spearman’s correlation analysis was used to explore the association of PI, TI, PI/TI, 25(OH) D3, and waist circumference with prediabetes. Odds ratios (OR) and 95% confidence intervals (CI) were calculated by logistic regression. Receiver-Operator Characteristic (ROC) curve was used to evaluate the risk of prediabetes.

**Results:**

Our study showed that FPI, 2hPI, FTI, 2hTI, FPI/FTI, and WC could enhance the risk of prediabetes (OR 1.034; OR 1.007; OR 1.005; OR 1.002; OR 3.577, OR 1.053, respectively; all *p*< 0.001). Stratified analyses indicated that FPI/FTI associated with an increased risk of prediabetes in men (OR 2.080, *p* = 0.042). FTI have a weak association with prediabetes risk in men and women (OR 0.987, *p* = 0.001; OR 0.994, *p* = 0.004, respectively). 2hPI could decrease prediabetes in women (OR 0.995, *p* = 0.037). Interesting, the sensitivity (86.0%) and AUC (0.942, *p*< 0.001) of combination (FPI+FTI+2hPI+2hTI+25(OH) D3+WC) were higher than the diagnostic value of these alone diagnoses. The optimal cutoff point of FPI, FTI, 2hPI, 2hTI, 25(OH) D3, and WC for indicating prediabetes were 15.5 mU/l, 66.5 mU/l, 71.5 mU/l, 460.5 mU/l, 35.5 ng/ml, and 80.5 cm, respectively. What’s more, the combination (FPI+FTI+2hPI+2hTI+25(OH) D3+WC) significantly improved the diagnostic value beyond the alone diagnoses of prediabetes in men and women (AUC 0.771; AUC 0.760, respectively).

**Conclusion:**

The FPI, 2hPI, FTI, 2hTI, FPI/FTI, and WC significantly associated with an increased risk of prediabetes. The combination of FPI, FTI, 2hPI, 2hTI, 25(OH) D3, and WC might be used as diagnostic indicators for prediabetes.

## Introduction

Diabetes mellitus (DM) is one of the most important chronic non-infectious diseases in the world, which has a serious impact on people's lives. China holds the largest number of DM patients in the world. According to IDF statistics in 2013, the number of patients with DM aged 20–79 years in China is about 98 million, which is expected to grow to 143 million by 2035 [1]. Type 2diabetes mellitus (T2DM) is one of the major diabetes. Prediabetes also knew as impaired glucose regulation (IGR), is a statue of abnormal glucose metabolism between normal glucose metabolism and diabetes. The epidemiological survey in 2010 showed that the number of patients with diabetes and prediabetes in China increased to 114 million and 493 million respectively, being accountable for 11.6% and 50.1% of adults [2]. The prevalence rate of prediabetes in the same population is usually higher than diabetes. In addition, people with prediabetes risk factors are most likely to develop diabetes than the general population [3]. Therefore, the intervention measures on the risk factors of prediabetes should be strengthened to control and reduce the occurrence of diabetes as far as possible.

Human proinsulin (PI), the precursor of true insulin (TI), which is comprised of TI and C peptide. PI is synthesized and secreted by pancreatic β cells and is mainly metabolized in the kidney. Only a few PI was released into the blood on physiological conditions, while pancreatic β cells can release PI leading to increased PI in blood under pathological conditions. Roder et al. revealed that the increased PI/TI can be used as a reflection of subclinical β cell dysfunction [4]. Another study noticed that PI/TI positively associated with type-2 diabetes [5]. Thus, PI/TI is an available factor to estimate the degree of pancreatic β cell dysfunction. And the enhanced PI/TI is used as an indicator for predicting diabetes risk.

25(OH) D3 (VD3), a kind of steroid hormone, act as a crucial role in the occurrence of diabetes by protecting pancreatic *β* cells and increasing insulin resistance of myocardial cells [6, 7]. A study indicated that the plasma VD3 level was closely associated with the pathogenesis of diabetes mellitus [8]. At present, most studies have found that VD3 negatively related to the risk of diabetes [9–11]. In addition, an increased study suggested that 25-hydroxyvitamin D3 can be used as one of the biochemical markers for predicting diabetes [12–14].

Waist circumference (WC) is a traditional body surface measurement index for evaluating central obesity which makes a high predictive value for the incidence of diabetes [15, 16].

In summary, several previous studies have focused on the correlation between PI, TI, PI/TI, VD3, WC, and glucose metabolism in DM. However, there is not any survey combined with the above five indicators to screen the high-risk population of diabetes. Therefore, this cross-sectional study aimed to investigate the correlation between PI, TI, PI/TI, VD3, WC, and prediabetes adults in Hainan Han Population. We further assessed the diagnostic value of PI, TI, PI/TI, VD3, WC, and their combinations in diagnosing prediabetes. Our present study would give any available information for the prevention and management of diabetes.

## Materials and methods

### Study subjects

In the current study, we selected 1662 residents aged ≥ 18 years old who participated in the National Diabetes Prevalence Survey of the Chinese Medical Association in 2017 to carry out a 75-g oral glucose tolerance test (OGTT). We divided the participants into the healthy group and the prediabetes group according to the ADA diagnostic criteria for prediabetes in 2010 [17]. Prediabetes was defined as 100 mg/dl (5.6 mmol/L) ≤ FPG < 126 mg/dl (7.0 mmol/L) or OGTT 2h PG 140 mg/dl (7.8 mmol/L) ≤ 2hPG < 200 mg/dl (11.1 mmol/L) or 5.7% ≤ HbA1C < 6.4% [17]. All subjects must conform to the following exclusion criteria: 1) Patients with diabetes. 2) Patients with malignant tumors, severe liver and kidney diseases, and metabolic osteopathy. This study was approved by the Ethics Committee of Hainan Affiliated Hospital of Hainan Medical University, and all research experiments were performed depending on the standard protocol of Helsinki’s Declaration. Each individual was informed of the purpose of the sample collection and received written informed consent from them before the study.

### Anthropometry and metabolic variables

The body weight, height, blood pressure, and waist circumference of all subjects were measured on an empty stomach in the morning. Body mass index (BMI) was calculated by the formula of weight / height-squared (m^2^). Fasting venous blood samples were collected for detecting metabolic parameters. The level of fasting plasma glucose (FPG), fasting true insulin (FTI), high-density lipoprotein cholesterol (HDL-C), low-density lipoprotein cholesterol (LDL-C), total cholesterol (TC), triglyceride (TG) and blood uric acid (BUA) were detected by an automatic biochemical analyzer. The concentration of 25-hydroxyvitamin D3 (25(OH) D3) was checked with a non-radioactive Enzyme Immunoassay kit. The hemoglobin A1c (HbA1c) was determined by high-performance liquid chromatography (HPLC).

### Data analysis

All statistical analysis of this study was performed by the SPSS version 17.0 software. Statistical tests were two-tailed and *p*< 0.05 indicated statistical significance. All variables except for gender were examined for non-normal distribution. Differences in age and clinical characteristics between healthy and prediabetes groups were analyzed by using the Rank sum test. The difference of gender between the cases and controls was analyzed by *χ*^*2*^ test. The interrelationship among FPI, 2hPI, FTI, 2hTI, 25(OH) D3, WC, and 2hPG was analyzed by spearman’s correlation analysis. We further analyzed the correlation of the indicators with prediabetes stratified by gender. We also assessed the association of FPI, 2hPI, FTI, 2hTI, 25(OH) D3, and WC with prediabetes through univariate and multivariate logistic regression analyses with adjusting for age, sex, WC, BMC, FPG, TG, TC, LDL-C, HDL-C, HbA1c, FPI, 2hPI, FTI, 2hTI, and 2hPI/2hTI. Odds ratios and 95% confidence intervals were calculated to evaluate the association. The relationship between indicators and the risk of prediabetes in men and women was carried out by using univariate logistic regression analysis after adjustment for age, WC, BMC, FPG, TG, TC, LDL-C, HDL-C, HbA1c, FPI, 2hPI, FTI, 2hTI, and 2hPI/2hTI. Tests for interaction between indicators and gender were performed using likelihood ratio tests. The receiver operating characteristic curve (ROC) was applied to evaluate the diagnostic performance of PI, TI, PI/TI, 25(OH) D3, WC, and the optimal cutoff points also were calculated in diagnosing prediabetes. The optimal cutoff points, sensitivity, and specificity values for diagnosing prediabetes were determined according to the Youden index. The area under the ROC curve (AUC) was used to compare the ability of PI, TI, PI/TI, 25(OH) D3, and WC to diagnose the risk of prediabetes [18].

## Results

### Basic characteristics of study populations

As is shown in Table 1, our current study consisted of 615 prediabetes (2hPG ≥ 7.8 mmol/L) and 1047 non-prediabetes (2hPG < 7.8 mmol/L). The average age was 44.89 ± 13.10 years old in a non-prediabetes group and 53.29 ± 12.24 years in the prediabetes group. There were significant statistical differences in age, gender, WC, BMC, FPG, TG, TC, LDL-C, HDL-C, HbA1c, FPI, 2hPI, FTI, 2hTI, and 2hPI/2hTI between non-prediabetes and prediabetes group (All *p* < 0.05). However, there was no difference in 25(OH) D3 and FPI/FTI between the two groups (*p* = 0.088, *p* = 0.166; respectively).

**Table 1:** General characteristics of the study subjects by 2hPG level.

Variables	2hPG		*p*
	< 7.8 mmol/L (n = 1047)	≥ 7.8 mmol/L (n = 615)	
Age, years	44.89 ± 13.10	53.29 ± 12.24	< 0.001^a^
Gender			0.003^b^
Men	354	253	
Women	693	362	
WC (cm)	79.26 ± 9.96	84.40 ±11.17	< 0.001^a^
BMI (kg/m^2^)	23.26 ± 3.76	24.63 ± 3.27	< 0.001^a^
FPG (mmol/l)	5.06 ± 0.55	6.20 ± 2.11	< 0.001^a^
TG (mmol/L)	1.68 ± 1.37	2.33 ± 2.49	< 0.001^a^
TC (mmol/l)	5.30 ± 1.04	5.62 ± 1.17	< 0.001^a^
LDL-C (mmol/l)	2.89 ± 0.81	3.07 ± 0.87	< 0.001^a^
HDL-C (mmol/l)	1.53 ± 0.39	1.46 ± 0.36	< 0.001^a^
BUA	346.07 ± 86.18	376.73 ± 91.52	< 0.001^a^
HbA1c (%)	5.45 ± 0.54	6.16 ± 1.31	< 0.001
25(OH) D3 (ng/ml)	37.31 (36.67–37.97)	38.29 (37.40–39.17)	0.088^a^
FPI (mU/l)	12.00 (11.43–12.57)	17.24 (15.84–18.64)	< 0.001^a^
2hPI (mU/l)	55.12 (52.18–58.05)	74.34 (69.61–79.07)	< 0.001^a^
FTI (mU/l)	61.63 (58.56–64.71)	75.23 (71.11–79.35)	< 0.001^a^
2hTI (mU/l)	375.76 (357.30–394.21)	700.65 (656.06–745.24)	< 0.001^a^
FPI/FTI	0.23 (0.22–0.25)	0.30 (0.29–0.33)	0.166^a^
2hPI/2hTI	0.19 (0.18–0.20)	0.15 (0.14–0.17)	< 0.001^a^

*p*^a^ were obtained by Rank sum test. *p*^*b*^ was calculated by *X*^2^ test. *p* < 0.05 indicates statistical significance.

WC, Waist circumference; BMI, Body mass index; FPG, Fasting plasma glucose; HDL-C, High-density lipoprotein cholesterol; LDL-C, Low-density lipoprotein cholesterol; TC, total cholesterol; TG, Triglyceride; BUA: Blood uric acid; HbA1c: Glycosylated hemoglobin; 25(OH) D3, 25-hydroxyvitamin D3; FPI, Fasting Proinsulin; 2hPI, 2h Proinsulin; FTI, Fasting True insulin; 2hTI, 2h True insulin.

### The correlation analysis between indicators and prediabetes

We used the spearman’s correlation analysis to explore the correlation between FPI, 2hPI, FTI, 2hTI, FPI/FTI, 2hPI/2hTI, 25(OH) D3, WC, and prediabetes, and the results were listed in Table 2. Our findings showed that patient with prediabetes positively correlated with FPI (r = 0.172, *p* < 0.001), 2hPI (r = 0.197, *p* < 0.001), FTI (r = 0.199, *p* < 0.001), 2hTI (r = 0.439, *p* < 0.001), FPI/FTI (r = 0.058, *p* = 0.018), 25(OH) D3 (r = 0.055, *p* = 0.026) and WC (r = 0.305, *p* < 0.001), respectively. While 2hPI/2hTI was negatively related to prediabetes (r = -0.066, *p* = 0.008).

**Table 2:** The Spearman’s correlation analysis between indicators and patients with prediabetes.

	r	*p*
FPI	0.172	< 0.001
2hPI	0.197	< 0.001
FTI	0.199	< 0.001
2hTI	0.439	< 0.001
FPI/ FTI	0.058	0.018
2hPI/2hTI	-0.066	0.008
25(OH) D3	0.055	0.026
WC	0.305	< 0.001

FPI, Fasting Proinsulin; 2hPI, 2h Proinsulin; FTI, Fasting True insulin; 2hTI, 2h True insulin; 25(OH) D3, 25-hydroxyvitamin D3; WC, Waist circumference.

*p* values were calculated by the Spearman’s correlation analysis and *p*< 0.05 indicates statistical significance.

We further analyzed the correlation of the indicators with prediabetes stratified by gender (Table 3). In men, FPI (r = 0.155, *p* < 0.001), 2hPI (r = 0.228, *p* < 0.001), FTI (r = 0.105, *p* = 0.010), 2hTI (r = 0.444, *p* < 0.001), FPI/ FTI (r = 0.141, *p* = 0.001), 2hPI/2hTI (r = -0.085, *p* = 0.039), and WC (r = 0.219, *p* < 0.001) were significantly correlated with prediabetes. Besides, FPI (r = 0.177, *p* < 0.001), 2hPI (r = 0.171, *p* < 0.001), FTI (r = 0.259, *p* < 0.001), 2hTI (r = 0.437, *p* < 0.001), and WC (r = 0.363, *p* < 0.001) were positively associated with prediabetes in women.

**Table 3:** The correlation analysis between indicators and prediabetes stratified by gender.

Indicators	Men		Women	
	r	*p*	r	*p*
FPI	0.155	< 0.001	0.177	< 0.001
2hPI	0.228	< 0.001	0.171	< 0.001
FTI	0.105	0.010	0.259	< 0.001
2hTI	0.444	< 0.001	0.437	< 0.001
FPI/ FTI	0.141	0.001	-0.014	0.659
2hPI/2hTI	-0.085	0.039	-0.057	0.067
25(OH) D3	0.001	0.976	0.060	0.053
WC	0.219	< 0.001	0.363	< 0.001

FPI, Fasting Proinsulin; 2hPI, 2h Proinsulin; FTI, Fasting True insulin; 2hTI, 2h True insulin; 25(OH) D3, 25-hydroxyvitamin D3; WC, Waist circumference.

*p* values were calculated by the Spearman’s correlation analysis and *p*< 0.05 indicates statistical significance.

### Association between the indicators and risk of prediabetes

We further investigated the relationship between FPI, 2hPI, FTI, 2hTI, 2hPI/2hTI, FPI/FTI, 25(OH) D3, WC, and risk of prediabetes (Table 4). We used univariate andmultivariate regression models with adjusting for WC, BMC, FPG, TG, TC, LDL-C, HDL-C, HbA1c, FPI, 2hPI, FTI, 2hTI, and 2hPI/2hTI to evaluate these associations. In univariate analyses, we found that FPI (OR = 1.034, 95% CI = 1.025–1.044, *p* < 0.001), 2hPI (OR = 1.007, 95% CI = 1.005–1.009, *p* < 0.001), FTI (OR = 1.005, 95% CI = 1.003–1.008, *p* < 0.001), 2hTI (OR = 1.002, 95% CI = 1.002–1.003, *p* < 0.001) and WC (OR = 1.053, 95% CI = 1.041–1.064, *p* < 0.001) were significantly correlated with prediabetes. In the multivariate model, FPI (OR = 1.034, 95% CI = 1.018–1.050, *p* < 0.001), 2hTI (OR = 1.002, 95% CI = 1.002–1.003, *p* < 0.001), FPI/FTI (OR = 3.577, 95% CI = 1.970–6.498, *p* < 0.001), and WC (OR = 1.055, 95% CI = 1.043–1.067, p < 0.001) showed the strongest association with a risk of prediabetes, while PTI (OR = 0.992, 95% CI = 0.988–0.996, *p* < 0.001) and 2hPI/2hTI (OR = 0.072, 95% CI = 0.028–0.181, *p* < 0.001) was a protective role.

**Table 4:** The risk factors for prediabetes.

Characteristics	B	S.E.	Wald	OR (95% CI)	*p*
Univariate analysis					
FPI	0.034	0.005	52.140	1.034 (1.025–1.044)	< 0.001
2hPI	0.007	0.001	46.897	1.007 (1.005–1.009)	< 0.001
FTI	0.005	0.001	23.711	1.005 (1.003–1.008)	< 0.001
2hTI	0.002	0.000	159.758	1.002 (1.002–1.003)	< 0.001
FPI/ FTI	0.333	0.170	3.837	1.396 (1.000–1.949)	0.050
2hPI/2hTI	-0.198	0.221	0.808	0.820 (0.532–1.246)	0.369
25(OH) D3	0.008	0.005	3.150	1.008 (0.999–1.018)	0.076
WC	0.051	0.006	82.405	1.053 (1.041–1.064)	< 0.001
Multivariate analysis					
FPI	0.033	0.008	17.994	1.034(1.018–1.050)	< 0.001
2hPI	-0.002	0.002	1.903	0.998(0.994–1.001)	0.168
FTI	-0.008	0.002	14.772	0.992(0.988–0.996)	< 0.001
2hTI	0.002	0.000	74.680	1.002(1.002–1.003)	< 0.001
FPI/ FTI	1.275	0.305	17.520	3.577 (1.970–6.498)	< 0.001
2hPI/2hTI	-2.638	0.473	31.060	0.072 (0.028–0.181)	< 0.001
25(OH) D3	0.003	0.005	0.303	1.003 (0.993–1.013)	0.582
WC	0.053	0.006	83.784	1.055 (1.043–1.067)	< 0.001

OR, odds ratio; CI, confidence interval.

*p* values were calculated by univariate and multivariate logistic regression analysis after adjustment for age, sex, WC, BMC, FPG, TG, TC, LDL-C, HDL-C, HbA1c, FPI, 2hPI, FTI, 2hTI, and 2hPI/2hTI.

*p*< 0.05 indicates statistical significance.

The relationship between the indicators and risk of prediabetes in men and women was summarized in Table 5. Our findings showed that FPI (OR 1.036, *p* = 0.001), FTI (OR 0.987, *p* = 0.001), 2hTI (OR 1.003, *p* < 0.001), FPI/ FTI (OR 2.080, *p* = 0.042), and WC (OR 1.042, *p* = 0.001) were significantly related to the risk of prediabetes in men. And FPI (OR 1.040, *p* < 0.001), 2hPI (OR 0.995, *p* = 0.037), FTI (OR 0.994, *p* = 0.004), 2hTI (OR 1.002, *p* < 0.001), and WC (OR = 1.047, *p* < 0.001) were strongly associated with prediabetes risk in women. Besides, no significant interaction was observed (all *p*-interaction > 0.10). These results suggested that these indicators might be used as independent risk factors for prediabetes.

**Table 5:** The risk factors for prediabetes stratified by gender.

Characteristics	B	S.E.	Wald	OR (95% CI)	*p*	*p*-interaction
Men						
FPI	0.036	0.011	10.225	1.036 (1.014–1.059)	0.001	0.43
2hPI	-0.003	0.002	1.745	0.997 (0.993–1.001)	0.186	0.29
FTI	-0.013	0.004	11.604	0.987 (0.980–0.995)	0.001	0.16
2hTI	0.003	0.000	47.431	1.003 (1.002–1.004)	< 0.001	0.58
FPI/ FTI	0.732	0.361	4.124	2.080 (1.026–4.216)	0.042	0.47
2hPI/2hTI	-0.011	0.385	0.001	0.990 (0.465–2.105)	0.978	0.17
25(OH) D3	0.011	0.008	1.665	1.011 (0.994–1.027)	0.197	0.13
WC	0.041	0.012	11.374	1.042 (1.017–1.067)	0.001	0.54
Women						
FPI	0.039	0.011	12.917	1.040 (1.018–1.063)	< 0.001	0.22
2hPI	-0.005	0.002	4.337	0.995 (0.991–1.000)	0.037	0.41
FTI	-0.006	0.002	8.303	0.994 (0.989–0.998)	0.004	0.95
2hTI	0.002	0.000	68.587	1.002 (1.002–1.003)	< 0.001	0.64
FPI/ FTI	0.080	0.266	0.092	1.084 (0.644–1.825)	0.762	0.82
2hPI/2hTI	0.244	0.500	0.237	1.276 (0.479–3.400)	0.626	0.71
25(OH) D3	-0.001	0.009	0.012	0.999 (0.982–1.016)	0.076	0.31
WC	0.045	0.008	29.870	1.047 (1.030–1.064)	< 0.001	0.66

OR, odds ratio; CI, confidence interval.

*p* values were calculated by univariate logistic regression analysis after adjustment for age, WC, BMC, FPG, TG, TC, LDL-C, HDL-C, HbA1c, FPI, 2hPI, FTI, 2hTI, and 2hPI/2hTI.

*p*< 0.05 indicates statistical significance.

The values of *p*-interaction were calculated by likelihood ratio tests and *p* < 0.10 indicates statistical significance.

### The diagnostic value of FPI, 2hPI, FTI, 2hTI, 2hPI/2hTI, FPI/FTI, 25(OH) D3, and WC for prediabetes

**Fig 1:**
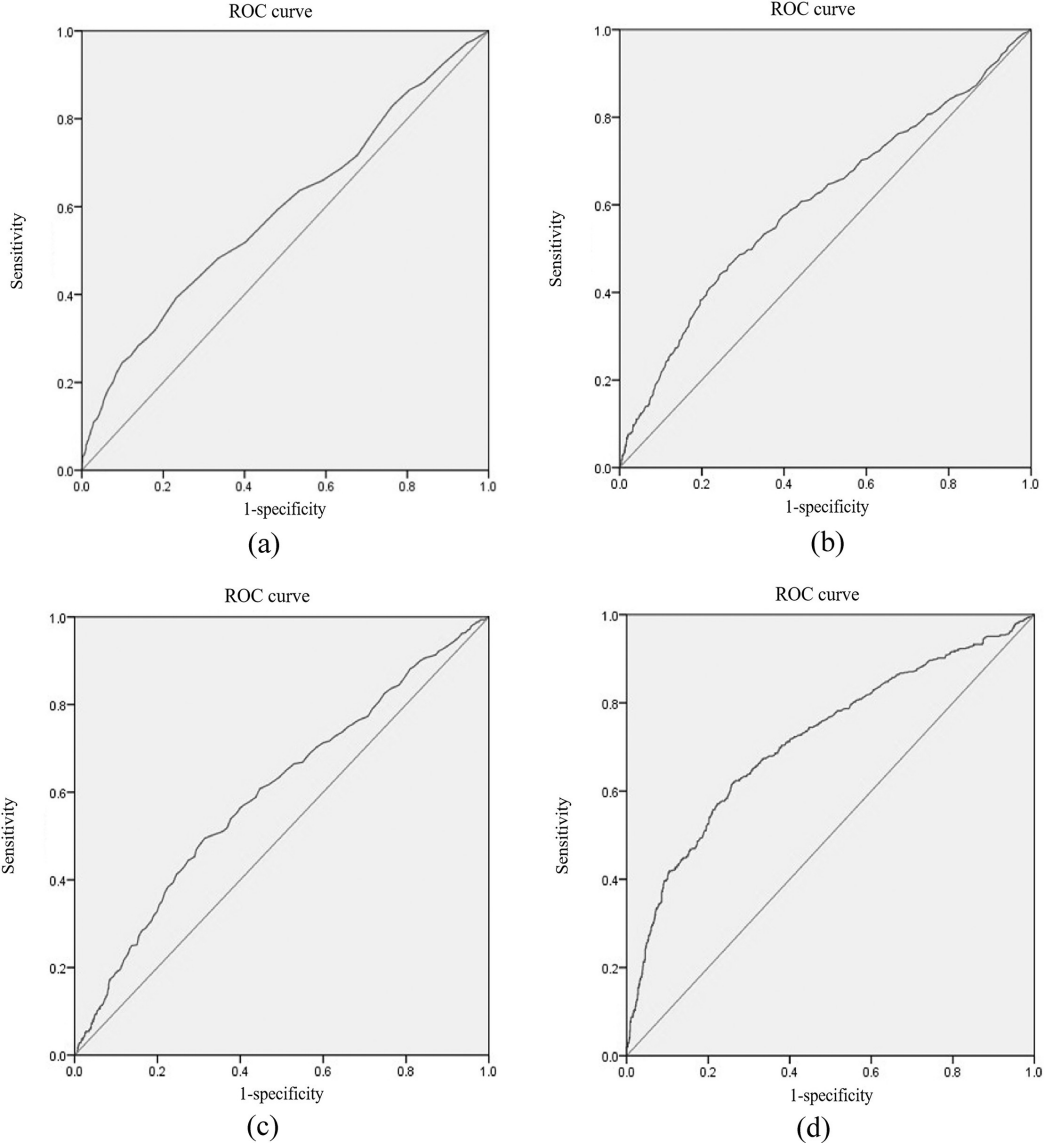
ROC curve analysis for predicting diabetes. (a) Fasting proinsulin concentration; (b) 2h proinsulin concentration; (c) Fasting insulin concentration; (d) 2h insulin concentration.

**Fig 2:**
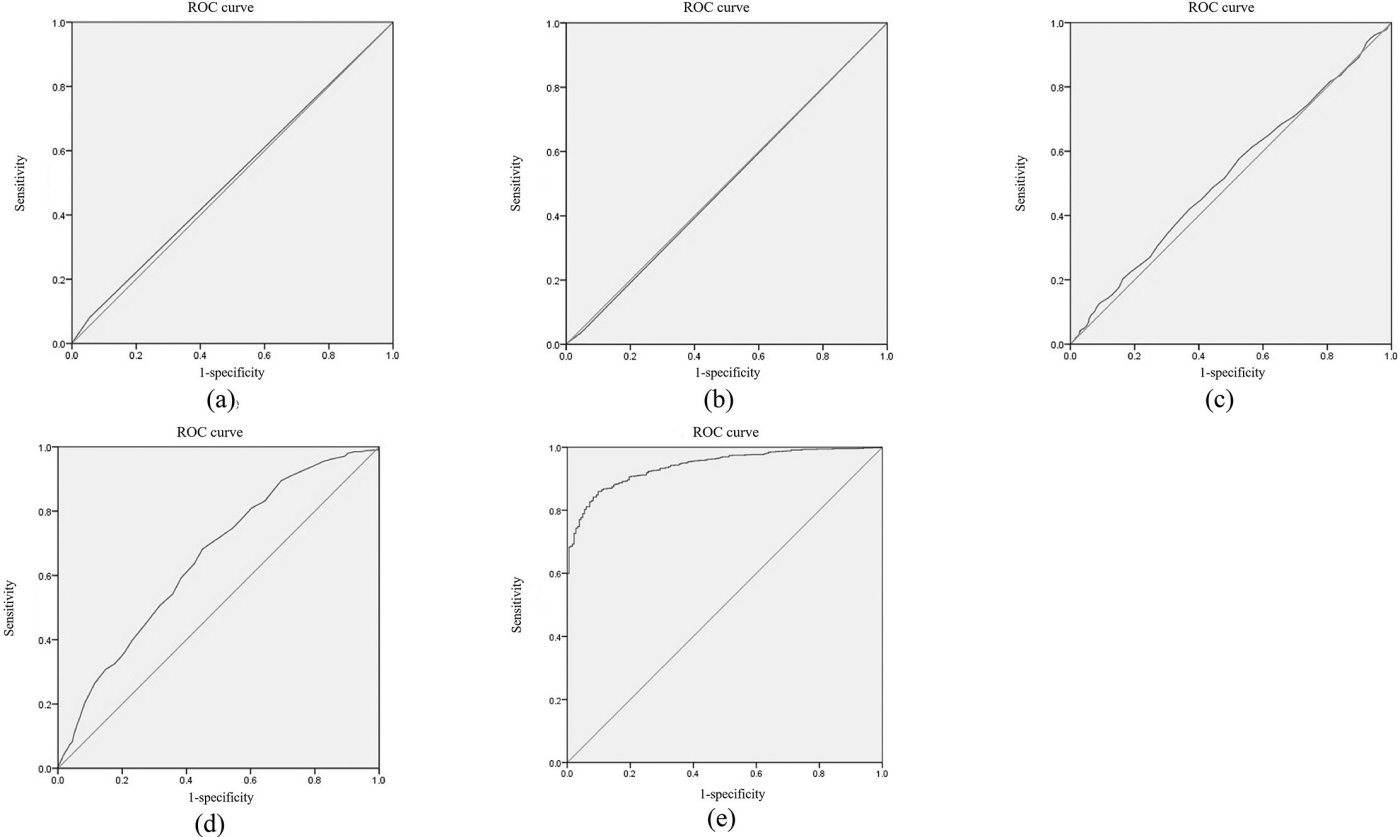
ROC curve analysis for predicting diabetes. (a) Fasting proinsulin: insulin ratio; (b) 2h proinsulin: insulin ratio; (c) 25(OH) D3 concentration; (d) Waist circumference; (e) FPI+FTI+2hPI+2hTI+25(OH) D3+WC.

ROC curve analysis was used to analyze the prognostic accuracy of FPI, 2hPI, FTI, 2hTI, 2hPI/2hTI, FPI/FTI, 25(OH) D3, WC, and their combinations in diagnosing prediabetes. The results are shown in Table 6, Figs 1 and 2. We found that the area under curve (AUC) of the combinations (FPI + FTI + 2hPI + 2hTI + 25(OH) D3 + WC) was 0.942 (95% CI = 0.93–0.95, *p* < 0.001), which the values of sensitivity and specificity were 86.0% and 90.2%, respectively. The sensitivity and AUC were larger than the diagnostic value of these alone diagnoses.

**Table 6:** Analysis of the effect of different indicators on the diagnosis of prediabetes.

	Cutoff	AUC (95% CI)	*p*	Sensitivity (%)	Specificity (%)	Youden index
FPI	15.5 mU/l	0.591 (0.56–0.62)	< 0.001	39.3	76.8	0.161
2hPI	71.5 mU/l	0.603 (0.57–0.63)	< 0.001	44.1	75.7	0.198
FTI	66.5 mU/l	0.599 (0.57–0.63)	< 0.001	49.5	68.6	0.181
2hTI	460.5 mU/l	0.715 (0.69–0.74)	< 0.001	61.9	73.9	0.358
FPI/FTI	0.5	0.513 (0.48–0.54)	0.387	8.0	94.5	0.250
2hPI/2hTI	1.5	0.495 (0.47–0.52)	0.725	0.3	99.7	0
25(OH) D3	35.5 ng/ml	0.525 (0.50–0.55)	0.090	57.7	47.5	0.52
WC	80.5 cm	0.652 (0.63–0.68)	< 0.001	68.2	55.0	0.232
FPI+FTI+2hPI+2hTI+25(OH) D3+WC	-	0.942 (0.93–0.95)	< 0.001	86.0	90.2	0.762

AUC, Area under a curve.

The value of cutoff, AUC, sensitivity, specificity, and Youden index were calculated by ROC analysis. *p*< 0.05 indicates statistical significance.

When stratified by gender, we observed that the diagnostic value of the combinations (FPI + FTI + 2hPI + 2hTI + 25(OH) D3 + WC) was significantly better than these alone diagnoses in prediabetes both men (AUC = 0.771, *p* < 0.001) and women (AUC = 0.760, *p* < 0.001) (Table 7).

**Table 7:** The effect of indicators on the diagnosis of prediabetes stratified by gender.

	Cutoff	AUC (95% CI)	*p*	Sensitivity (%)	Specificity (%)	Youden index
Men						
FPI	20.5 mU/l	0.585 (0.54–0.63)	< 0.001	33.7	81.9	0.157
2hPI	68.5 mU/l	0.628 (0.58–0.67)	< 0.001	55.7	67.5	0.232
FTI	67.5 mU/l	0.557 (0.51–0.60)	0.017	44.6	66.3	0.109
2hTI	454.0 mU/l	0.723 (0.68–0.77)	< 0.001	63.5	73.7	0.372
FPI/FTI	0.5	0.534 (0.48–0.58)	0.158	13.5	93.1	0.067
2hPI/2hTI	1.5	0.489 (0.44–0.54)	0.639	0	1	0.001
25(OH) D3	51.50 ng/ml	0.514 (0.47–0.56)	0.571	26.3	78.6	0.049
WC	89.50 cm	0.617 (0.63–0.70)	< 0.001	46.1	71.2	0.173
FPI+FTI+2hPI+2hTI+25(OH) D3+WC	-	0.771(0.73–0.81)	< 0.001	59.5	81.6	0.411
Women						
FPI	15.5 mU/l	0.587 (0.55–0.62)	< 0.001	34.8	81.9	0.167
2hPI	71.5 mU/l	0.581 (0.54–0.62)	< 0.001	37.8	78.7	0.166
FTI	66.5 mU/l	0.626 (0.59–0.66)	< 0.001	52.4	70.1	0.225
2hTI	516.5 mU/l	0.713 (0.68–0.75)	< 0.001	56.5	78.9	0.356
FPI/FTI	4.0	0.497 (0.46–0.53)	0.869	0	100	0.003
2hPI/2hTI	1.5	0.496 (0.46–0.53)	0.829	0	100	0.001
25(OH) D3	35.50 ng/ml	0.507 (0.47–0.54)	0.690	44.9	58.9	0.037
WC	80.50 cm	0.668 (0.63–0.70)	< 0.001	57.5	68.6	0.261
FPI+FTI+2hPI+2hTI+25(OH) D3+WC	-	0.760(0.73–0.79)	< 0.001	64.7	77.4	0.420

AUC, Area under a curve.

The value of cutoff, AUC, sensitivity, specificity, and Youden index were calculated by ROC analysis. *p*< 0.05 indicates statistical significance.

## Discussion

In this cross-sectional study, we recruited 1662 individuals to investigate the correlation between FPI, 2hPI, FTI, 2hTI, 2hPI/2hTI, FPI/FTI, 25(OH) D3, WC, and risk of prediabetes in Hainan Han adults. We found that there was a significantly positive correlation between FPI, 2hPI, FTI, 2hTI, FPI/FTI, 25(OH) D3, WC, and 2hPG, while 2hPI/2hTI negatively correlated with 2hPG. Moreover, FPI, 2hPI, FTI, 2hTI, WC, and FPI/FTI were significantly associated with the risk of prediabetes. For men, FPI, 2hPI, FTI, 2hTI, FPI/ FTI, and WC were positively correlated with prediabetes, while 2hPI/2hTI negatively related to prediabetes. For women, FPI, 2hPI, FTI, 2hTI, and WC were positively associated with prediabetes. More interesting, our findings revealed that the sensitivity and AUC of the combinations (FPI + FTI + 2hPI + 2hTI + 25(OH) D3 + WC) were much greater than the diagnostic value of these alone diagnoses. The combination of FPI, FTI, 2hPI, 2hTI, 25(OH) D3, and WC significantly improved the diagnostic value beyond the alone diagnoses of prediabetes in men and women. It suggested that FPI, FTI, 2hPI, 2hTI, 25(OH) D3, and WC may be used as diagnostic indicators for prediabetes.

As previous studies have demonstrated that prediabetes was an impaired glucose regulation state which mainly caused by the pancreatic β cell dysfunction with insulin secretion and hepatic insulin resistance (IR) cell damage [19, 20]. Besides, people with prediabetes have a high risk of developing diabetes mellitus, especially T2DM [21]. Numerous researches have indicated that PI, TI, 25(OH) D3, and WC were associated with the occurrence of T2DM [5, 22–25].

In our study, the association between PI, TI, and risk of prediabetes was explored in Hainan Han adults by using spearman’s correlation analysis and logistic regression analysis. We found that FPI, FTI, and FPI/FTI ratio positively correlated with prediabetes. FPI, FTI, and FPI/FTI ratio were significantly associated with an increased risk of prediabetes. These results are in agreement with Pradhan’s findings in middle-aged women which showed that FTI can be used for a long-term risk predictor, FPI and FPI/FTI have a strong predictive role in the rapid development to T2DM [5]. In general, it seems that FPI and FTI could be a candidate risk factor in the diagnosis of prediabetes. Besides, we also observed that 2hPI/2hTI negatively relates to prediabetes. And the significant relationship was found between 2hPI/2hTI and prediabetes risk. In general, it seems that FPI, FTI, FPI/FTI, and 2hPI/2hTI could be a candidate risk factor in the diagnosis of prediabetes.

We further investigate the correlation between 25(OH) D3 and prediabetes. It found that 25(OH) D3 was positively related to prediabetes, but it cannot use for risk factors. Aleksandra et al. also indicated that 25(OH) D3 was not an association with the risk of prediabetes in US civilians, however, they showed 25(OH) D3 significantly increased risk of T2DM [26]. However, there was a study that revealed that the lower level of 25(OH) D3 has significantly enhanced the risk of T2DM among Japanese adults, especially in the prediabetes population [27]. These diverse results may be due to the regional difference among them.

Several previous studies suggested that WC is a better predictor for hypertension and T2M, however, there is no report on prediabetes [28, 29]. Thus, we also detected the correlation between WC and prediabetes. We found that WC significantly associates with increased risk of prediabetes. The present findings are consistent with several previous studies indicating that WC may be an appropriate indicator for diagnosing T2DM [30–32].

The ROC curve was finally performed to evaluate the diagnostic value of PI, TI, 25(OH) D3, and WC for prediabetes. Interestingly, the sensitivity (86.0%) and AUC (area = 0.942) of combination (FPI + FTI + 2hPI + 2hTI + 25(OH) D3 + WC) were higher than the diagnostic value of these alone diagnoses.

Our present study has some limitations. Firstly, the sample size of participants is relatively small for an epidemiological survey. More sample size could provide further definitive evidence. Secondly, we haven’t explored the correlations stratified by age, and so on, which can be investigated in further work. Despite its limitations, the study certainly provided very useful evidence for a diagnostic indicator of prediabetes.

## Conclusions

In summary, our study indicated that FPI, 2hPI, FTI, 2hTI, 2hPI/2hTI, FPI/FTI, 25(OH) D3, and WC were correlated with prediabetes. FPI, 2hPI, FTI, 2hTI, FPI/FTI, and WC may be used for risk factors in prediabetes. The diagnostic value of the combination (FPI + FTI + 2hPI + 2hTI + 25(OH) D3 + WC) was higher than that of these alone diagnoses. The optimal cutoff point of FPI, FTI, 2hPI, 2hTI, 25(OH) D3, and WC for indicating prediabetes were 15.5 mU/l, 66.5 mU/l, 71.5 mU/l, 460.5 mU/l, 35.5 ng/ml, and 80.5 cm, respectively. Our findings will give useful information for the diagnosis and management of prediabetes.

## Supporting information

S1 Database (XLSX)
